# Association of Pre-Pregnancy Obesity and COVID-19 with Poor Pregnancy Outcome

**DOI:** 10.3390/jcm12082936

**Published:** 2023-04-18

**Authors:** Sladjana Mihajlovic, Dejan Nikolic, Biljana Milicic, Milena Santric-Milicevic, Natalya Glushkova, Zhansaya Nurgalieva, Milan Lackovic

**Affiliations:** 1 University Hospital “Dragisa Misovic”, Heroja Milana Tepica 1, 11000 Belgrade, Serbia; 2 Faculty of Medicine, University of Belgrade, 11000 Belgrade, Serbia; 3 Department of Physical Medicine and Rehabilitation, University Children’s Hospital, 11000 Belgrade, Serbia; 4 Department of Medical Statistics and Informatics, School of Dental Medicine, University of Belgrade, 11000 Belgrade, Serbia; 5 Institute of Social Medicine, Faculty of Medicine, University of Belgrade, 11000 Belgrade, Serbia; 6 Center-School of Public Health and Health Management, Faculty of Medicine, University of Belgrade, 11000 Belgrade, Serbia; 7 Faculty of Medicine and Health Care, Al-Farabi Kazakh National University, Almaty 050044, Kazakhstan

**Keywords:** pregnancy, COVID-19, obesity, co-morbidities, maternal outcomes, pregnancy outcomes, clinical characteristics

## Abstract

Background and Objectives: During the COVID-19 pandemic, a possible overlap of obesity and COVID-19 infection has raised concerns among patients and healthcare professionals about protecting pregnant women from developing a severe infection and unwanted pregnancy outcomes. The aim of this study was to evaluate the associations of body mass index with clinical, laboratory, and radiology diagnostic parameters as well as pregnancy complications and maternal outcomes in pregnant patients with COVID-19. Materials and Methods: Clinical status, laboratory, and radiology diagnostic parameters and pregnancy outcomes were analyzed for pregnant women hospitalized between March 2020 and November 2021 in one tertiary-level university clinic in Belgrade, Serbia, due to infection with SARS-CoV-2. Pregnant women were divided into the three sub-groups according to their pre-pregnancy body mass index. For testing the differences between groups, a two-sided *p*-value <0.05 (the Kruskal–Wallis and ANOVA tests) was considered statistically significant. Results: Out of 192 hospitalized pregnant women, obese pregnant women had extended hospitalizations, including ICU duration, and they were more likely to develop multi-organ failure, pulmonary embolism, and drug-resistant nosocomial infection. Higher maternal mortality rates, as well as poor pregnancy outcomes, were also more likely to occur in the obese group of pregnant women. Overweight and obese pregnant women were more likely to develop gestational hypertension, and they had a higher grade of placental maturity. Conclusions: Obese pregnant women hospitalized due to COVID-19 infection were more likely to develop severe complications.

## 1. Introduction

Obesity in the female population is a public health threat given the noticeable rise in the global incidence of overweight adult women, from 29.8% in 1980s to nearly 40% in 2010s [1,2]. It has become a universal healthcare challenge for the management of pregnant woman, requiring more knowledge for the prevention of gestational diabetes mellitus, gestational hypertension and pre-eclampsia, fetal macrosomia, stillbirth, and pre-term delivery [3,4]. During the COVID-19 pandemic, a possible overlap of obesity and COVID-19 infection has raised concerns among patients and healthcare professionals about protecting pregnant women from developing a severe form of infection and poor pregnancy outcomes. Obesity is usually associated with the complexity and disruption of various mechanisms, including changes in respiratory function [5] and the immune system, and imposes significant risk for developing severe complications of COVID-19 infection [6], particularly during pregnancy [7]. Previous knowledge on the associated increased rates of morbidity and mortality among overweight and obese pregnant women during the pandemics of varicella zoster virus, cytomegalovirus as well as influenza in 1918, 1957, and 2009 [8,9,10,11] alerted that similar threats to the health and the well-being of the mother, fetus, and newborn can happen during the COVID-19 pandemic. More studies are needed to understand the impact of the COVID-19 pandemic on pregnant women [12,13].

Independently, COVID-19 infection in pregnancy is a risk factor for some of the most severe conditions in perinatology, including preeclampsia, pre-term birth, low birth weight, and stillbirth [14]. Prolonged and even fatal inflammation lies at the interface of complex interferences of obesity, viral infection, and immunity changes [15]. In the general population, an obesity paradox is seen for moderate obesity and inflammation, resulting in lower mortality rates in patients suffering from heart and kidney failure, acute respiratory distress syndrome (ARDS) [16], and pneumonia [17]. However, among pneumonia cases caused by SARS-CoV-2, severe forms of COVID-19 were correlated with increased body mass index values [18]. This phenomenon among pregnant women requires more attention, and in this study, we tried to detect the association between overweight and obesity and clinical and pregnancy outcomes and to test the assumption of obesity paradox among pregnant patients with COVID-19.

Previous findings in pregnant patients with obesity and COVID-19 indicate more frequent thromboembolism and preeclampsia, higher risk of nosocomial infections [15], more frequent admission to intensive care units and more frequent use of invasive and non-invasive oxygen therapy [19], and maternal death [16]. However, a number of researchers have found a similar severity of infection with COVID-19 in pregnant and non-pregnant women [20,21,22,23,24] and that pregnant women suffer less frequently from COVID-19 than from infection with other corona viruses [25]. Consequently, the diverse findings in the literature actually call attention to the drivers of poor maternal outcomes among obese pregnant women with COVID-19 infection as challenges for every community, and more evidence is needed to inform and prepare health professionals for the management of such cases.

Globally, more than 500 million cases of infection and nearly 7 million deaths have been directly associated with COVID-19 infection [26]. According to official data, there have been more than 2 million recorded cases of COVID-19 infection in Serbia, with more than 16,000 deaths caused by the SARS-CoV-2 infection [27]. No national official reports disclose number of infected or deceased pregnant woman, raising the necessity for more research to explore COVID-19 disease treatment and progression among pregnant women presenting at our hospitals. Furthermore, concerns of new COVID-19 pandemic waves, as well as sequelae following the infection, oblige us to provide new insights and maintain caution [28,29]. Therefore, the aim of this study is to present the clinical, laboratory, and radiological diagnostic status and pregnancy outcomes of COVID-19-infected pregnant patients in Serbia. In addition, in this study, we assessed the diagnostics and outcome differences in pregnant women with COVID-19 according to their body mass index. This study will contribute to the current knowledge on prenatal and postnatal considerations for pregnant patients with COVID-19 [30]. The results of this study could be used to inform national stakeholders and to provide new insights for case management in obstetrics to prevent complications and poor outcomes.

## 2. Methods

### 2.1. Study Design

This was a retrospective case series study, and it included pregnant women who needed hospitalization due to SARS-CoV-2 infection (COVID-19 disease). Patients in this study were infected with SARS-CoV-2 and hospitalized between March 2020 and November 2021 at the referral center for COVID-19-infected pregnant patients in Serbia, University Hospital “Dr. Dragisa Misovic” in Belgrade, Serbia. The clinical status of COVID-19 infection was detected by a nasopharyngeal swab and confirmed by real-time polymerase chain reaction (RT-PCR). Exclusion criteria for this study were negative PCR test for SARS-CoV-2 and multiple pregnancies. The Institutional Review Board approved this study (Decision No. 01-8816, 3 August 2020).

### 2.2. Study Population Description

#### 2.2.1. Study Participants

The patients in this study were referred from other gynecological clinics and COVID hospitals in Serbia to our center due to infection with COVID-19 during pregnancy. To study obesity and COVID-19 infection as predecessors of poor maternal and pregnancy outcomes, patients were divided into three groups based on their pre-pregnancy body mass index (BMI) values and World Health Organization (WHO) recommendations [31]. BMI was calculated according to the subject’s weight and height; weight calculated in kilograms was divided by the square of height in meters. Data regarding pre-pregnancy BMI values were obtained from primary health center records.

#### 2.2.2. Admission Criteria for Hospitalization

At admission, the initial examination was conducted based on the adapted version of the Modified Early Obstetric Warning Score (MEOWS) [32]. Accordingly, we evaluated six parameters: respiratory rate, peripheral blood oxygen saturation, arterial blood pressure, pulse, body temperature, and level of consciousness. We counted the number of breaths per minute (chest-rising breath followed by one chest-falling exhalation that was counted as one full breath); a pulse oximeter was used to asses blood oxygen saturation and heart rate; body temperature was measured with a thermometer in degrees Celsius; blood pressure was monitored manually; and level of consciousness was determined with the Awake, Verbal, Pain, Unresponsive (AVPU) scale [33].

Respiratory rate between 21 and 30 breaths per minute, peripheral blood oxygen saturation <95%, heart rate between 100 and 120 per minute, systolic blood pressure >140 mmHg, diastolic >90 mmHg, and body temperature >38 °C were considered as criteria for the hospitalization of our patients. Transfer to the intensive care unit (ICU) was indicated when respiratory rate was <10 or >30 per min, oxygen blood saturation <90%, heart rate >120 per min, systolic blood pressure >180 mmHg, diastolic >110 mmHg, body temperature >38 °C, and the patient was unresponsive to treatment as well as exhibiting disorientation and loss of consciousness and verbal communication.

### 2.3. Study Variables (Clinical Data)

For three groups of patients, we collected data for seven sets of study variables, a total of 46 variables. The first set included pregnancy-related clinical endpoints such as the number of days from the onset of COVID-19 symptoms to the start of hospitalization, length of hospital stay, duration of intensive care in days, presence of drug-resistant nosocomial infection, and duration of mechanical ventilation in days. In the second set were clinical parameters related to COVID-19, including fever, cough, difficult breathing, headache, loss of smell, loss of taste, diarrhea, ARDS, pulmonary embolism, multiple organ failure, maternal death, and peak of deterioration. The third and fourth sets contained data on diagnostic imaging (computed tomography (CT) and CT score) and therapy (antibiotics, corticosteroids, and low-molecular-weight heparin (LMWH)). The fifth and sixth sets were used to describe eight characteristics of pregnant women and seven characteristics of newborns. Finally, the seventh set contained the standard nine biochemical parameters.

We collected medical history data to describe characteristics of pregnant women such as anthropometric parameters, previous chronic and family history for cardiovascular disease (CVD), gestational hypertension and diabetes mellitus, primary thrombophilia, and anemia from patient records. Gestational hypertension was defined as an arterial blood pressure value above 140/90 mmHg after two separate measurements and after 20 weeks of gestation [34]. The diagnosis of glucose intolerance onset in pregnancy, gestational diabetes mellitus, was established according to the American Diabetes Association’s recommendations [35]. There were no recorded cases of pre-gestational diabetes mellitus.

Anamnesis data included the number of days from the onset of symptoms of COVID-19 to hospitalization, as well as whether the patient had a loss of smell and taste, cough, headache, diarrhea, fever, and difficult breathing. CT was performed depending on the patient’s clinical presentation and laboratory testing results, e.g., for all patients in the ICU. The chest CT score by Li and associates [36] was applied to assess the degree and severity of lung involvement. The CT score varied from 0 to 25 depending on the sum of individual lobar involvement.

In addition, the patient’s medical records contained data on the duration of hospitalization, duration of intensive care, days of oxygen therapy required, number of prescribed antibiotics, use of corticosteroid therapy and LMWH, presence/absence of acute respiratory distress syndrome (ARDS), nosocomial infection, pulmonary embolism, multiple organ failure (MOF), peak worsening of health status since the onset of symptoms (peak of deterioration), and maternal death. The criteria for ARDS were based on the Berlin definition of ARDS [37]. Lung and cardiovascular impairment, liver and kidney injury, coagulation disorders, and neurological manifestations indicated MOF [38]. The diagnosis of pulmonary embolism was established using the YEARS diagnostic algorithm [39].

From the obstetric records, we took data on the outcome of the pregnancy (live birth, intrauterine fetal demise (stillbirth), or miscarriage), gestational age at delivery (pre-term, full-term, and post-term newborn), and the newborn baby’s Apgar score in the first and the fifth minute of life. According to gestational age at delivery, pre-maturity was defined as gestational age <37 gestational weeks and post-maturity as pregnancies lasting ≥42 gestational weeks [40]. All pre-term deliveries were performed therapeutically and due to the deterioration of maternal condition.

At admission, ultrasonography included the assessment of the amniotic fluid index (AFI), the grade of the placenta maturity according to Grannum classification, and the dynamics of fetal growth. Fetal growth >90th percentile was used to define large-for-gestational-age (LGA) fetuses, and intrauterine fetal growth restriction (IUGR) was defined according to the Delphi consensus [41].

### 2.4. Study Variables (Biochemical Data)

Biochemical parameters included creatinine, urea, lactate dehydrogenase (LDH), alanine transaminase (ALT), aspartate transaminase (AST), iron, C-reactive protein (CRP), ferritin, procalcitonin, and D-dimer. Apart from at admission and discharge, laboratory diagnostics was performed every two to three days for all patients, except for patients in the ICU, whose laboratory results were collected daily.

### 2.5. Protocols for Managing COVID-19

The health authorities of Serbia issued a total of eleven different versions of the national protocol for the treatment of COVID-19 infection, which emphasized that multidisciplinary teamwork and an individualized therapeutic approach should be applied, considering the specificity of the patient’s health condition [42]. Accordingly, antibiotics were recommended for cases of likely or proven bacterial superinfection. A small number of patients were candidates for antiviral drugs, and adverse reactions or side effects associated with the use of antiviral drugs were not reported. All hospitalized patients were treated with low-molecular-weight heparin in prophylactic doses, while therapeutic doses were applied in cases of suspected or proven development of venous thrombosis. Prevention of cytokine storm in cases of severe maternal infection usually involved the introduction of methylprednisolone after the fourth day of disease onset. During the observed period, immunomodulatory therapy, immunoglobulins, convalescent plasma, and neutralizing monoclonal antibodies were not used.

### 2.6. Statistical Analysis

Results are presented as absolute (n) and relative (%) numbers, mean values (MVs) and standard deviation (SD). Additionally, 95% confidence intervals (CIs) were introduced for continuous variables. Statistical significance was set at *p* < 0.05. All statistical tests were performed with SPSS Statistics V.22.0. Comparisons between tested groups of patients were performed using the Kruskal–Wallis test and the ANOVA test for continuous variables and the chi square test for categorical variables. The Wilcoxon signed-rank test was used to compare tested variables at admission with those at discharge in the defined patient groups. The Mann–Whitney U test was used for comparisons between two tested groups of continuous variables, while the chi square test was used for categorical variables.

## 3. Results

Our study included 192 subjects: 53 (27.6%) normal weight, 92 (47.9%) overweight, and 47 (24.5%) obese pregnant women. There were more obese and overweight pregnant women with positive family history of cardiovascular disease (*p* = 0.028), with gestational hypertension (*p* = 0.007), and stillbirth (*p* = 0.008) (Table 1). In addition, the highest average grade of placental maturity was found among the obese patients (*p* = 0.041) (Table 1).

Average duration of hospitalization and days in ICU were the longest for obese pregnant women with COVID-19 (*p* = 0.045 and *p* = 0.039, respectively) (Table 2). Furthermore, more so than others, obese pregnant women had multi-organ failure (*p* = 0.049) and drug-resistant nosocomial infection (*p* = 0.049). Cases of maternal mortality were more often recorded in obese pregnant women (*p* = 0.013). In addition, corticosteroids (*p* = 0.030) and antibiotic therapy (*p* = 0.028) were prescribed more often in obese pregnant women (Table 2). Only two obese pregnant women (i.e., 4.7% of all obese patients) had a pulmonary embolism.

Compared to their counterparts, obese pregnant women with COVID-19 had the lowest values of ferritin (*p* = 0.003) and procalcitonin at admission (*p* < 0.001) and of iron (*p* = 0.003) and procalcitonin at discharge (*p* = 0.010) (Supplementary Table S1).

## 4. Discussion

In our study, over two-thirds of the pregnant patients with COVID-19 were overweight or obese. Obese patients had increased hospital and ICU durations, were more likely to develop multi-organ failure and drug-resistant nosocomial infections, were prescribed a higher number of antibiotics, and had increased probability of corticosteroid therapy application. Ultimately, they more frequently had lethal maternal outcomes and increased stillbirth rates. Overweight and obese patients were more likely to develop gestational hypertension, while obese patients had the highest grade of placental maturity.

As in other studies [43,44], we also found in the group of obese and overweight pregnant women a significant increase in the duration of hospitalization; days in ICU; and frequency of life-threatening complications such as nosocomial infections, pulmonary embolism, multi-organ failure, and ultimately maternal mortality. Even though initial signs and symptoms of infection, such as difficult breathing, cough, headache, loss of smell, and taste, did not differ significantly between the three compared groups of patients, they were observed more often in the group of patients with normal weight. The peak of deterioration was on average two days later in the group of obese patients. Medical professionals at triage points should carefully consider these differences as obese patients are significantly more likely to develop life-threatening complications despite a late onset of signs and symptoms of infection. Therefore, close monitoring of obese pregnant patients should be mandatory, and preventive hospitalization should be considered in cases where it is not possible to provide adequate supervision for outpatients.

Pregnancy itself leads to significant changes in the respiratory system and, in combination with the multifactorial influence of overweight and obesity, can lead to a decrease in functional residual capacity and consequently to reduced respiratory function [45]. This is one of the explanations for the observed differences in requirements, as well as in the average duration of mechanical ventilation. Normal-weight and overweight patients spent an average of a day and a half on the various modes of mechanical ventilation regimens, and obese patients had more than twice the average duration of mechanical ventilation, nearly four days. Obese patients, unlike normal and overweight patients, were the only group of patients diagnosed with pulmonary embolism (PE). The highest levels of D-dimer were observed in the obese patients, which were almost double the value of that in patients with normal weight. Combined obesity and SARS-CoV-2 infection [46], as well as obesity alone [47], are associated with increased rates of PE in pregnant and post-partum women. CT is recommended for the diagnosis of PE [48], and it was performed more frequently in obese patients. CT was performed in less than a quarter of normal-weight and overweight patients, while more than a third of patients in the obese group had this type of diagnostics. The average CT scores in all three groups were comparable.

The number of prescribed antibiotics and corticosteroid therapy use significantly differed between those compared, in contrast to LMWH, probably due to its routine administration as recommended by the Royal College of Obstetrics and Gynecologists [49]. Inflammation and obesity often go hand in hand [50], which is probably the reason why corticosteroid therapy was more often used in obese patients. On the other hand, the suppression of inflammatory reactions could allow the progression of bacterial infections and lead to the use of more antibiotics in the obese group of patients compared to the other two groups.

Procalcitonin (PCT) is a widely used biomarker associated with bacterial infection incorporated in the routine differentiation of infection origin, and it has proven its utility in predicting secondary bacterial infection in patients with COVID-19 [51]. The highest PCT and CRP levels were recorded in the obese group of patients, followed by the group of overweight patients. The group of obese patients was treated with a higher number of antibiotics, and the probability of nosocomial infection was significantly higher in this group. Given the vulnerability of the immune system in overweight and obese patients [52], these patients should undoubtedly be treated with more caution.

Studies have shown that SARS-CoV-2 is associated with worse maternal outcomes, but the direct link between COVID-19 infection and poor pregnancy outcome is still debatable [53]. In our case series, the stillbirth rate was more than 10 times higher in the obese group of patients compared to that in normal weight patients. In the obese group of patients, nearly 13% of pregnancies resulted in stillbirth. This raises questions about the quality of obstetric care and fetal monitoring since obesity alone increases susceptibility to pregnancy complications [54]. During the pandemic, an increase in the stillbirth rate reported in Austria supported the hypothesis that the combination of obesity and SARS-CoV-2 poses additional risks for stillbirth [55]. In our study, the range of pre-maturity was from 21.6% to 34.7%, with the highest rate in the group of patients with normal weight, which also had the highest percentage of live births. Iatrogenic pre-maturity associated with SARS-CoV-2 could lead to an increase in neonatal morbidity and mortality [56,57], but it seems necessary and justified for the prevention and reduction of stillbirth [40]. During the one of the most severe lockdowns in Australia, iatrogenic pre-maturity was significantly reduced, but pre-term stillbirths significantly increased [58], which emphasizes the relevance of regular antepartum fetal surveillance and adequate obstetric care. Maternal obesity can cause technical difficulties in fetal monitoring and assessment, contributing to the risk of stillbirth [59]. Similarly, placental dysfunction is a pregnancy complication commonly associated with intrauterine growth restriction and stillbirth [60]. During our initial fetal ultrasonographic evaluation with Granum’s classification, we assessed the highest grade of placental maturity in obese patients and a high grade in overweight patients. Despite classification arguments [61], these results coincide with the highest stillbirth rate in the group of obese patients. Given its clinical significance, we suggest using this ultrasonography marker in correlation with other parameters for deciding when and how to terminate the pregnancy and deliver a baby. Systematic reviews have confirmed that SARS-CoV-2 affects the placenta causing primarily vascular malformations, leading to the formation of intervillous thrombus, chorangiosis, and villitis [62]. Nevertheless, a universal pathomorphological pattern has not yet been established [63], which probably makes the power of ultrasonography and the decision-making process even more debatable in predicting these changes.

Obesity is certainly one of the universal risk factors for the development of arterial hypertension in all age groups, and the state of pregnancy is no exception. In our study, we found a significant difference in the incidence of positive family history for cardiovascular disease (CVD) and gestational hypertension in the obese group of patients, one of the most common and most dangerous co-morbidities in pregnancy [64]. In addition to obesity, advanced maternal age and chronic hypertension, diabetes, and asthma were risk factors for disease severity, according to a French study [65]. A strong association between obesity, pregnancy, and severity of disease was confirmed in a study from the United States [66]. Obesity-related co-morbidities are preventable, and pre-conception counseling could significantly improve the reduction of pre-pregnancy obesity and related comorbidities.

Anemia has shown a strong positive correlation with pregnancy [67], obesity [68], and COVID-19 [69]. Significant changes in iron metabolism in patients with severe COVID-19 were observed shortly after the pandemic’s onset [70]. Systematic reviews have found a significant difference in ferritin levels but not in other biomarkers of iron metabolism among critically ill patients [71]. Our study results confirm these findings in pregnant obese women as well. More precisely, in addition to PCT values, ferritin and iron values at hospital admission were laboratory parameters in which we identified a substantial difference between the three groups of pregnant women. Furthermore, a recently conducted meta-analysis revealed that, aside from well-known chronic and pregnancy-related comorbidities, anemia caries an increased risk for the severe form of the COVID-19 disease and poor maternal and pregnancy outcomes and pointed out the importance of this less commonly known risk factor [72].

This study has several strengths. The study participants were pregnant women with COVID-19 infection hospitalized in the reference hospital. Furthermore, the strength of this study is in the comprehensive analysis of 46 clinical, radiological, and laboratory parameters, with which it contributes internationally to the scarce literature in this field.

There were several limitations to this study. The studied population’s size in Serbia is unknown, thus limiting the findings’ representativeness. Despite their validity, this study’s results should not be generalized to other populations or hospitals with different protocols for treating pregnant women with COVID-19. This study’s design did not allow the identification of a causal relationship, but it explored the associations of poor pregnancy outcomes with obesity and COVID-19. The obesity paradox could not be confirmed among pregnant patients with COVID-19. Nevertheless, we found a later onset of signs and symptoms of infection and peak deterioration in obese women with COVID-19 compared to counterparts, probably explaining the longer hospital and ICU stays. At the same time, they were significantly more likely to develop life-threatening complications and have stillbirths. These results need to be confirmed in further research by considering new variables such as vaccinal status and changes in the treatment protocols. The important findings on nosocomial infections, stillbirths, and maternal deaths have also opened questions about the quality of health care, for which combined quantitative and qualitative research could be appropriate.

## 5. Conclusions

In this study, maternal and neonatal outcomes in women with COVID-19 and excessive weight were worse compared to those in women with a normal weight. Special attention must be given to monitoring clinical parameters that are indicators for the development of life-threatening complications such as pulmonary embolism and multi-organ failure. Antepartum fetal surveillance, ultrasonography evaluation, and timely intervention should prevent unfavorable pregnancy outcomes.

The healthcare of hospitalized obese pregnant women with COVID-19 has been a challenge for obstetricians and intensive care specialists globally due to a broad spectrum of clinical manifestations and serious complications. In that regard, this study’s findings should be used to inform treatment strategies and preventive measurements and to direct more attention to case management in obstetrics to prevent complications and poor outcomes.

## Figures and Tables

**Table 1:** Clinical features of pregnant women with COVID-19 by body mass index categories.

Variables		Body Mass Index (BMI)			*p*
		Normal Weight n = 53	Overweight n = 92	Obese n = 47	
**Patient’s characteristics and pregnancy-related complications**					
Age, MV ± SD (95% CI)		30.66 ± 4.98 (29.29–32.02)	30.51 ± 5.53 (29.36–31.66)	31.06 ± 5.42 (29.47–32.66)	0.847 ^c^
Positive family history for CVD, N (%)		11 (20.8%)	22 (23.9%)	20 (42.6%)	*0.028* ^b^
Gestational hypertension, N (%)		2 (3.8%)	9 (9.8%)	11 (23.4%)	*0.007* ^b^
Gestational diabetes mellitus, N (%)		2 (3.8%)	6 (6.5%)	6 (12.8%)	0.209 ^b^
Primary thrombophilia, N (%)		2 (3.8%)	8 (8.7%)	2 (4.3%)	0.413 ^b^
Anemia, N (%)		15 (28.3%)	36 (39.1%)	20 (42.6%)	0.283 ^b^
Gestational age at labor, median (variance)		38 weeks and 5 days (1678.5)	39 weeks and 1 day (288.7)	38 weeks and 1 day (541.8)	0.323 ^a^
Pregnancy outcome, N (%)	Live birth	49 (92.4%)	88 (95.65%)	41 (87.2%)	*0.008* ^b^
	Stillbirth	1 (1.9%)	4 (4.35%)	6 (12.8%)	
	Miscarriage	3 (5.7%)	0 (0%)	0 (0%)	
**Newborn’s characteristics**					
Live newborns, N (%)	Pre-term	17 (34.7%)	19 (21.6%)	11 (26.8%)	0.474 ^b^
	Term	32 (65.3%)	68 (77.3%)	30 (73.2%)	
	Post-term	0 (0%)	1 (1.1%)	0 (0%)	
Amniotic fluid index, median (variance)		120.00 (751.7)	120.00 (1141.4)	130 (2033.4)	0.139 ^a^
Placenta maturity grade, median (variance)		2.50 (0.9)	3.00 (0.8)	3.00 (0.5)	*0.041* ^a^
Intrauterine growth restriction, N (%)		3 (5.8%)	4 (4.5%)	1 (2.2%)	0.674 ^b^
Large for gestational age, N (%)		0 (0.0%)	2 (2.2%)	3 (6.5%)	0.128 ^b^
Apgar score 1st minute, median (variance)		9.00 (8.5)	9.00 (4.5)	9.00 (10.1)	0.500 ^a^
Apgar score 5th minute, median (variance)		10.00 (9.8)	10.00 (5.5)	10.00 (12.2)	0.807 ^a^

^a^ Kruskal–Wallis test; ^b^ chi square test; ^c^ ANOVA test; CVD—cardiovascular disease; MV—mean value; SD—standard deviation; CI—confidence interval.

**Table 2:** Differences in signs and symptoms of COVID-19 in pregnant patients according to their body mass index.

Variables	Body Mass Index (BMI)			*p*
	Normal Weight n = 53	Overweight n = 92	Obese n = 47	
**Clinical endpoints**				
Days from COVID-19 symptom onset to hospitalization initiation, median (variance)	3.00 (15.7)	3.00 (20.9)	4.00 (30.4)	0.283 ^a^
Length of hospital stay (ICU duration excluded) (days), median (variance)	4.00 (31.5)	5.00 (23.2)	6.00 (66.7)	*0.045* ^a^
ICU duration (days), median (variance)	0.00 (6.3)	0.00 (10.2)	0.00 (66.2)	*0.039* ^a^
Drug-resistant nosocomial infection, n (%)	1 (1.9%)	1 (1.1%)	4 (8.5%)	*0.049* ^b^
Number of patients on mechanical ventilation, n (%)	9 (17%)	13 (14.1%)	13 (27.7%)	0.143 ^b^
Duration of mechanical ventilation (days), median (variance)	0.00 (19.0)	0.00 (26.5)	0.00 (63.3)	0.095 ^a^
**Clinical status parameters related to COVID-19 infection**				
Body temperature >38 °C on admission, n (%)	28 (52.8%)	50 (54.3%)	29 (61.7%)	0.628 ^b^
Cough, n (%)	24 (45.3%)	40 (44.0%)	24 (51.1%)	0.723 ^b^
Difficult breathing, n (%)	13 (24.5%)	17 (18.5%)	9 (19.1%)	0.666 ^b^
Headache, n (%)	9 (17.0%)	12 (13.0%)	7 (14.9%)	0.809 ^b^
Smell loss, n (%)	21 (39.6%)	24 (26.1%)	12 (25.5%)	0.177 ^b^
Taste loss, n (%)	20 (37.7%)	22 (23.9%)	11 (23.4%)	0.152 ^b^
Diarrhea, n (%)	0 (0.0%)	5 (5.4%)	1 (2.1%)	0.175 ^b^
ARDS, n (%)	2 (3.8%)	4 (4.3%)	6 (12.8%)	0.104 ^b^
Pulmonary embolism, n (%)	0 (0.0%)	0 (0.0%)	2 (4.5%)	*0.039* ^b^
Multi-organ failure, n (%)	1 (1.9%)	1 (1.1%)	4 (8.5%)	*0.049* ^b^
Maternal death, n (%)	1 (1.9%)	1 (1.1%)	5 (10.6%)	*0.013* ^b^
Peak of deterioration (days), median (variance)	4.00 (17.4)	4.00 (17.5)	6.00 (30.8)	0.065 ^a^
**Diagnostic imaging**				
CT, n (%)	12 (22.6%)	20 (21.7%)	18 (38.3%)	0.088 ^b^
CT score, median (variance)	8.00 (53.8)	10.50 (27.8)	11.00 (57.2)	0.852 ^a^
**Therapy**				
Average number of antibiotics prescribed per patient, median (variance)	1.00 (1.6)	1.0 (1.5)	2.0 (3.3)	*0.028* ^a^
Number of patients with corticosteroid therapy, n (%)	9 (17.0%)	6 (6.5%)	10 (21.3%)	*0.030* ^b^
LMWH, n (%)	35 (66.0%)	59 (64.1%)	39 (83.0%)	0.062 ^b^

^a^ Kruskal–Wallis test; ^b^ chi square test; ICU—intensive care unit; ARDS—acute respiratory distress syndrome; MOF—multi-organ failure; CT—computerized tomography; LMWH—low-molecular-weight heparin; MV—mean value; SD—standard deviation; CI—confidence interval.

## Data Availability

All data are presented in this study. Original data are available on reasonable request from the corresponding author.

## Supplementary Materials

The following supporting information can be downloaded at: https://www.mdpi.com/article/10.3390/jcm12082936/s1, Table S1: Biochemical parameters at admission and discharge of pregnant patients with COVID-19 according to their body mass index.
