# Supplementary material for: Association of Pre-Pregnancy Obesity and COVID-19 with Poor Pregnancy Outcome
Source: J Clin Med. 2023 Apr 18;12(8):2936. doi: 10.3390/jcm12082936 (PMC10144693; doi:10.3390/jcm12082936)
Supplement: Supplementary file 1 [file jcm-12-02936-s001.zip › jcm-2232945-supplementary.pdf]

Supplementary material S1.

Supplementary Table S1. Biochemical parameters at admission and discharge of pregnant patients with COVID-19 according to their body mass index.

**Variables**	**Body mass index (BMI)**			**p [a]**
	**Normal weight n=53**	**Overweight n=92**	**Obese n=47**	
Creatinine at admission, Mean (Variance)	57.00 (2791.3)	55.50 (257.4)	55.50 (398.0)	0.989
Creatinine at discharge, Mean (Variance)	57.00 (4387.6)	54.50 (1907.7)	54.00 (115.7)	0.564
p [d]	0.472	0.243	0.052	
LDH at admission, Median (Variance)	216.00 (55312.6)	193.00 (7442.6)	204.00 (44131.4)	0.054
LDH at discharge, Median (Variance)	223.00 (23990.9)	201.50 (12988.5)	205.00 (8491.6)	0.315
p [d]	*0.033*	0.785	0.089	
ALT at admission, Median (Variance)	26.00 (2650.4)	20.00 (2946.8)	21.00 (2323.3)	0.101
ALT at discharge, Median (Variance)	36.00 (8868.9)	26.00 (1568.9)	33.50 (776.6)	0.062
p [d]	0.054	0.253	*0.003*	
AST at admission, Median (Variance)	28.00 (13585.9)	23.00 (1681.8)	26.00 (665.9)	0.169
AST at discharge, Median (Variance)	25.00 (1512.8)	24.00 (413.9)	27.00 (1245.1)	0.303
p [d]	0.774	0.359	0.591	
Iron at admission, Median (Variance)	10.00 (86.4)	10.00 (65.1)	8.00 (21.6)	0.140
Iron at discharge, Median (Variance)	12.00 (29.8)	12.00 (43.9)	9.50 (16.3)	*0.003*
p [d]	*0.048*	0.057	0.298	
CRP at admission, Median (Variance)	20.90 (2602.7)	32.90 (4488.9)	48.20 (2183.2)	0.110
CRP at discharge, Median (Variance)	16.40 (672.4)	17.20 (2054.8)	24.75 (1302.4)	0.201
p [d]	*0.005*	*<0.001*	*0.004*	

Ferritin at admission, Median (Variance)	72.00 (29362.5)	42.00 (53695.6)	50.00 (32519.4)	*0.003*
Ferritin at discharge, Median (Variance)	50.00 (82173.3)	40.00 (30197.9)	48.00 (13979.9)	0.248
p [d]	*0.006*	0.329	0.942	
Procalcitonin at admission, Median (Variance)	0.05 (0.04)	0.05 (3.36)	0.06 (2.49)	*<0.001*
Procalcitonin at at discharge, Median (Variance)	0.05 (0.002)	0.04 (1.08)	0.05 (0.08)	*0.010* [a]
p [d]	*0.005*	*0.002*	*<0.001*	
D-dimer at admission, Median (Variance)	2.50 (4.8)	2.60 (36.3)	2.26 (8.7)	0.872
D-dimer at discharge, Median (Variance)	1.53 (0.7)	1.35 (7.8)	2.62 (25.6)	0.884
p [d]	*<0.001*	*<0.001*	*<0.001*	

[a] Kruskal Wallis test; [d] Wilcoxon Signed Rank test; LDH – lactate dehydrogenase; ALT – alanine transaminase; AST – aspartate transaminase; CRP – C-reactive protein; MV -mean value; SD - standard deviation; CI - confidence interval.
